# A large-scale screening for the taiga tick, *Ixodes persulcatus*, and the meadow tick, *Dermacentor reticulatus*, in southern Scandinavia, 2016

**DOI:** 10.1186/s13071-019-3596-3

**Published:** 2019-07-09

**Authors:** Lene Jung Kjær, Arnulf Soleng, Kristin Skarsfjord Edgar, Heidi Elisabeth H. Lindstedt, Katrine Mørk Paulsen, Åshild Kristine Andreassen, Lars Korslund, Vivian Kjelland, Audun Slettan, Snorre Stuen, Petter Kjellander, Madeleine Christensson, Malin Teräväinen, Andreas Baum, Anastasia Isbrand, Laura Mark Jensen, Kirstine Klitgaard, René Bødker

**Affiliations:** 1 Department of Veterinary and Animal Sciences, Faculty of Health and Medical Sciences, University of Copenhagen, Frederiksberg, Denmark; 2 Department of Pest Control, Norwegian Institute of Public Health, Oslo, Norway; 3 Department of Virology, Norwegian Institute of Public Health, Oslo, Norway; 4 Department of Production Animal Clinical Sciences, Norwegian University of Life Sciences, Oslo, Norway; 5 Department of Natural Sciences, University of Agder, Kristiansand, Norway; 6 Sørlandet Hospital Health Enterprise, Research Unit, Kristiansand, Norway; 7 Department of Production Animal Clinical Sciences, Section of Small Ruminant Research, Norwegian University of Life Sciences, Sandnes, Norway; 8 Wildlife Ecology Unit, Department of Ecology, Swedish University of Agricultural Sciences, Grimsö, Sweden; 9 Department of Applied Mathematics and Computer Science, Technical University of Denmark, Lyngby, Denmark; 10 Department for Diagnostics and Scientific Advice, National Veterinary Institute, Technical University of Denmark, Lyngby, Denmark

**Keywords:** Taiga tick, *Ixodes persulcatus*, Siberian and Far Eastern tick-borne encephalitis, meadow tick, *Dermacentor reticulatus*, southern Scandinavia, range expansion

## Abstract

The taiga tick, *Ixodes persulcatus*, has previously been limited to eastern Europe and northern Asia, but recently its range has expanded to Finland and northern Sweden. The species is of medical importance, as it, along with a string of other pathogens, may carry the Siberian and Far Eastern subtypes of tick-borne encephalitis virus. These subtypes appear to cause more severe disease, with higher fatality rates than the central European subtype. Until recently, the meadow tick, *Dermacentor reticulatus*, has been absent from Scandinavia, but has now been detected in Denmark, Norway and Sweden. *Dermacentor reticulatus* carries, along with other pathogens, *Babesia canis* and *Rickettsia raoultii*. *Babesia canis* causes severe and often fatal canine babesiosis, and *R. raoultii* may cause disease in humans. We collected 600 tick nymphs from each of 50 randomly selected sites in Denmark, southern Norway and south-eastern Sweden in August–September 2016. We tested pools of 10 nymphs in a Fluidigm real time PCR chip to screen for *I*. *persulcatus* and *D. reticulatus*, as well as tick-borne pathogens. Of all the 30,000 nymphs tested, none were *I. persulcatus* or *D. reticulatus.* Our results suggest that *I. persulcatus* is still limited to the northern parts of Sweden, and have not expanded into southern parts of Scandinavia. According to literature reports and supported by our screening results, *D. reticulatus* may yet only be an occasional guest in Scandinavia without established populations.

**Letter to the Editor**

Tick-borne diseases pose a risk to both humans and animals [1–3], and there is a concern that the increase in incidence and geographical range reported over the last decades [4–8] may be an effect of climate change impacting vectors and their associated pathogens [9, 10]. In Europe, and especially Scandinavia, the main vector of disease-causing pathogens in humans, pets and other large mammals is the castor bean tick *Ixodes ricinus* [6, 7]. The closely related taiga tick, *Ixodes persulcatus*, has previously been limited to eastern Europe and northern Asia [11], but within the last 15 years, the species has expanded its range, both in eastern Europe [12, 13] but also towards western Europe [11, 12, 14]. *Ixodes persulcatus* was recorded in the western parts of Finland in 2004 [14] and 2008 [15], and in northern Sweden in 2015 [11]. *Ixodes persulcatus* may carry the Siberian and Far Eastern subtypes of the tick-borne encephalitis virus (TBEV) along with a range of other pathogens [11, 16, 17]. The Siberian and Far Eastern subtypes of TBEV have been reported to cause more severe symptoms than the European sub-type [17–19], although there is speculation that this may be due to other factors such as clinical alert and reporting [17, 19].

The meadow tick, *Dermacentor reticulatus*, is endemic to Europe [20], and is currently spreading to new geographical areas [20–22]. *Dermacentor reticulatus* was previously absent from Scandinavia [20], but has been found on migrating birds in Norway as early as 2003–2005 [23], and potentially in 2009, as *Babesia canis* was detected in a dog from the Oslo area that had not travelled abroad, indicating that *D. reticulatus* was present in the area [24]. In Sweden, single *D. reticulatus* has been identified in 2010 in the region of Skåne, in 2012 on a dog that had been abroad and then again two more times in the region of Skåne in 2017 [25]. In Denmark, *D. reticulatus* was found on a migrating golden jackal (*Canis aureus*) in 2017 [21], and again in 2018 on a dog that was returning from a trip to Slovakia with its owner [26]. *Dermacentor reticulatus* carries several pathogens presently absent in Scandinavia, but the most concerning involve *B. canis* and *Rickettsia raoultii*. *Babesia canis* causes canine babesiosis in dogs with a high risk of death [27]. *Rickettsia raoultii* poses a zoonotic health concern as it may cause disease in humans [21].

As a part of a large Scandinavian project, we randomly selected 30 sites in each of Denmark, southern Norway and south-eastern Sweden for tick collection in August and September 2016. Selection of the 90 sites was based on a stratification scheme with random sampling described in Kjær et al. [28]. Ticks were only analysed from sites where ≥ 600 nymphs could be collected, resulting in a total of 50 sites (Fig. 1).

**Fig. 1:**
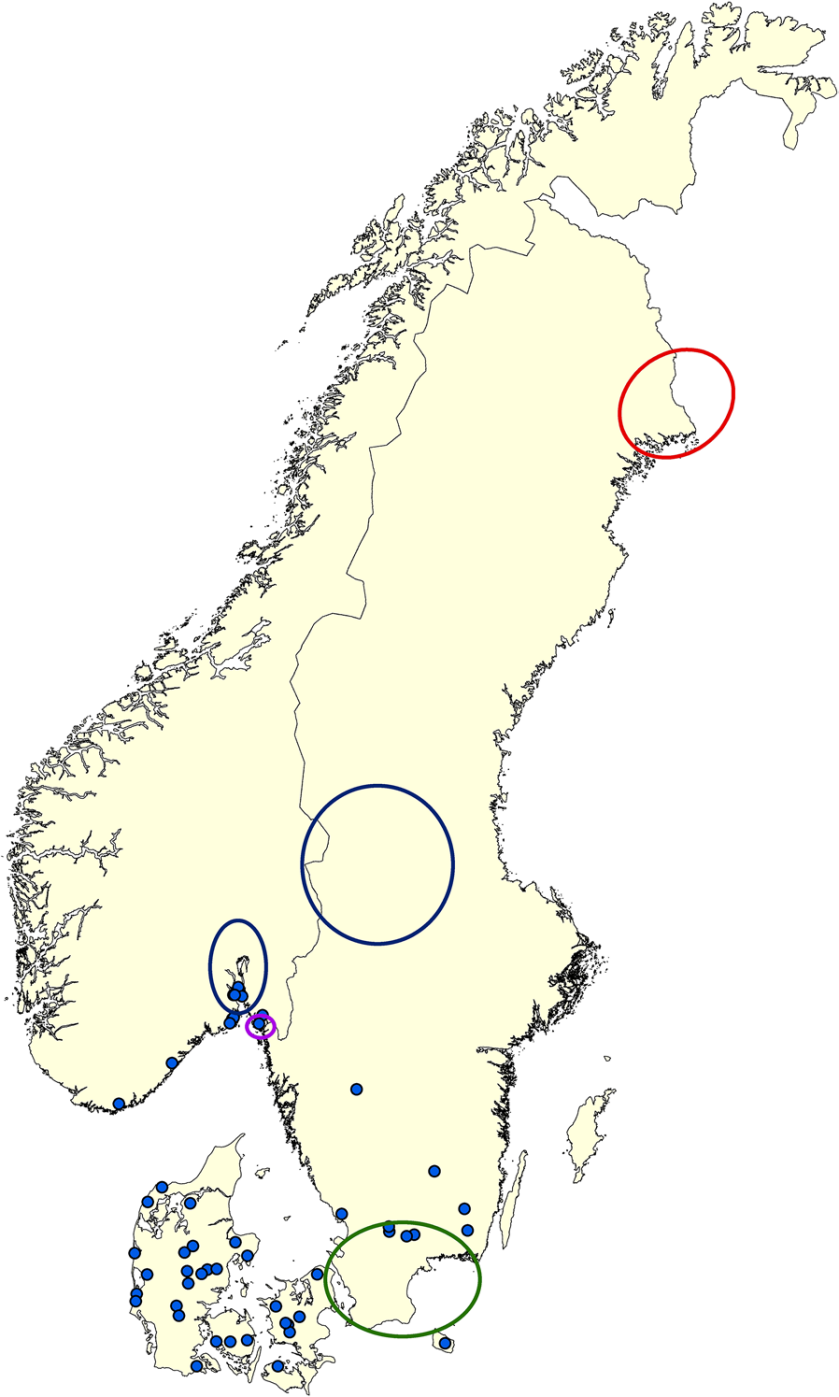
Map of southern Scandinavia with the 50 sample sites from 2016 depicted (blue dots). At each site, a minimum of 600 tick nymphs were collected. The red ellipse marks the area where *I. persulcatus* was recorded in 2015 by Jaenson et al. [11]. The blue ellipses are where *D. reticulatus*/*B. canis* was found associated with dogs [24, 25], the magenta ellipse is where *D. reticulatus* was found on birds [23] and the green ellipses is where *D. reticulatus* has been found in nature [25]

We morphologically examined the 30,000 ticks to ensure that they were all nymphs. We aggregated 30,000 collected nymphs into 60 pools of 10 for each site and used the BioMark real-time PCR system (Fluidigm, San Francisco, California, USA) for high-throughput microfluidic RT-PCR. The method is thoroughly described in Klitgaard et al. [29] and Michelet et al. [8]. Along with 18 different pathogens, we simultaneously screened each pool for presence of *D. reticulatus*, *I. persulcatus* and *I. ricinus,* as described and validated by Michelet et al. [8]. The Fluidigm chip has been used for surveillance of tick-borne pathogens and exotic tick species on both flagged ticks and on ticks removed from imported animals in Denmark since 2014. The chip has previously detected *D. reticulatus* on a migrating golden jackal [21].

We found that of the 30,000 nymphs tested, all pools tested positive for *I. ricinus*, and none for *I. persulcatus* or *D. reticulatus*. Using simple probability theory, we calculated a measure of “freedom from *I. persulcatus/D. reticulatus*”, using the binomial theorem:

$$DC = 1 - \left( {1 - prev} \right)^{N}$$

where *DC* is the degree of certainty (here 95%), *prev* is the proportion of *I. persulcatus/D. reticulatus*, and *N* is the sample size, here either 600 per site or 30,000 in total.

With this equation, we assume that if *I. persulcatus/D. reticulatus* constitute a proportion higher or equal to *prev* in all nymphs collected and the PCR is 100% sensitive in pool sizes of 10, we can then be 95% certain that we would detect at least one positive pool. With 600 ticks per site and all pools negative, we are therefore 95% certain that the proportion of *I. persulcatus/D. reticulatus* at each given site was lower than 0.5%, given the reasonable assumption that the 600 nymphs represent a random sample drawn from a much larger population at the site. Likewise, if the 30.000 nymphs collected in total were a random sample from the entire area, we would be 95% certain that the proportion of *I. persulcatus/D. reticulatus* would be lower than 0.01%. Therefore, if the two species are individually introduced by e.g. migrating birds to the region, they constitute less than one out of 10,000 flagged nymphs. However, if the two species are not just randomly introduced individuals but instead have become established breeding populations then they are likely to have a spatially clustered distribution in the area. With small clusters the probability of detecting a cluster by screening 50 sites is just 5.8% at a 95% certainty level, assuming the proportion of the species in a cluster is high enough to be detected with a sensitivity of 100% when 600 nymphs are tested per site. Thus, the existence of spatially limited clusters of locally breeding *I. persulcatus* or *D. reticulatus* in the area cannot be excluded with reasonable certainty, despite the large number of nymphs analysed.

Although there is no evidence for an increased northward distribution of permanent viable populations of *I. ricinus* in Norway [30], studies from Sweden have found *I. ricinus* to have expanded northwards compared to historical data [6], possibly due to climate change [9, 10]. Thus, a potential spread of *I. persulcatus* further south in Scandinavia and establishment of *D. reticulatus* within the Scandinavian region could also be expected. Further tick surveillance studies in Scandinavia should acknowledge the possibility of *I. persulcatus/D. reticulatus* becoming established further in this region, and thus the possibility of infections with the Siberian and Far Eastern subtypes of TBEV, *B. canis*, *R. raoultii* and other pathogens related to these two tick species. It may be advisable to carry out targeted surveillance by flagging at sites with reported cases of *B. canis* in dogs and Siberian and Far Eastern subtypes of TBE in humans without recent travel histories. Alternatively, it may be recommendable to initiate citizen science projects [31] as local breeding populations of *I. persulcatus* and *D. reticulatus* will be difficult to detect by random surveillance. Our results suggest that *I. persulcatus* and *D. reticulatus* may not be established in southern Scandinavia.

## Data Availability

The datasets analysed during the current study are available from the corresponding author on reasonable request.
